# Modulation of Doxorubicin Intracellular Accumulation and Anticancer Activity by Triterpene Glycoside Cucumarioside A_2_-2

**DOI:** 10.3390/md17110597

**Published:** 2019-10-23

**Authors:** Ekaterina Menchinskaya, Tatiana Gorpenchenko, Alexandra Silchenko, Sergey Avilov, Dmitry Aminin

**Affiliations:** 1 G.B. Elyakov Pacific Institute of Bioorganic Chemistry, Far-Eastern Branch of the Russian Academy of Science, Prospect 100-letya Vladivostoka, 159, 690022 Vladivostok, Russia; sialexandra@mail.ru (A.S.); avilov-1957@mail.ru (S.A.); daminin@piboc.dvo.ru (D.A.); 2 Federal Scientific Center of the East Asia Terrestrial Biodiversity, Far-Eastern Branch of the Russian Academy of Science, Prospect 100-letya Vladivostoka, 159, 690022 Vladivostok, Russia; gorpenchenko@biosoil.ru; 3 Department of Biomedical Science and Environmental Biology, Kaohsiung Medical University, 100, Shih-Chuan 1st Road, Kaohsiung 80708, Taiwan

**Keywords:** cucumarioside A_2_-2, doxorubicin, ehrlich ascites carcinoma, MDR, sea cucumbers, triterpene glycosides

## Abstract

The effect of treatment of Ehrlich ascites carcinoma (EAC) cells with multidrug resistance by holothurian triterpene glycoside, cucumarioside A_2_-2 (CA_2_-2) was evaluated. Calcein-AM efflux assay and doxorubicin (DOX) uptake and retention measurement in cancer cells, as well as determination of DOX cytotoxic and anticancer effects were applied. Treatment of EAC cells with CA_2_-2 (0.01–0.1 μM) blocked Calcein-AM and DOX efflux from cancer cells and increased the accumulation and cytotoxicity of DOX in EAC cells. Moreover, pre-treatment of mice with EAC by CA_2_-2 (10 μg/kg/5 days, intraperitoneal injection (*i.p.*)), then transplantation of tumor cells into fresh animals and subsequent treatment of these mice with DOX (2 mg/kg/3 days *i.p.*) significantly increased average life span (ALS) of mice bearing a tumor and therefore boosted the antitumor effect of doxorubicin in vivo.

## 1. Introduction

The resistance of tumor cells to many toxic antitumor drugs, which are used for chemotherapy of cancer and are dissimilar in chemical structure and mechanism of action, has long been a known phenomenon called multidrug resistance (MDR). Cells that have MDR or acquire it during chemotherapy become resistant to the action of drugs and the destruction or inhibition of their proliferation requires the use of chemotherapeutic cytostatics such as cyclophosphamide, doxorubicin, vinblastine, cisplatin and fluorouracil in doses so large that they often cause unwanted toxic effects. The most common MDR mechanism is the activation of transmembrane transport proteins that remove various substances from the cell. The main protein of this type is P-glycoprotein (P-gp) [1]. P-gp acts as a pump which, using the energy of ATP, can pump a wide variety of substances from the cell including a variety of antitumor cytostatics and cytotoxins that freely penetrate through the cell membranes by diffusion. The use of new effective MDR blockers would help solve the problem arising from the use of chemotherapy. 

It is known that there are drugs that help to overcome MDR of tumors, such as verapamil, cyclosporin A, nifedipine and its derivatives, amiodarone, trifluoperazine, quinine and others [2]. The disadvantage of these compounds is the presence of serious side effects in the doses necessary to enhance the action of antitumor drugs. In particular, verapamil, an effective inhibitor of P-gp, causes heart failure and brain damage, and cyclosporin A has immunosuppressive activity. In this regard, the search for MDR inhibitors among natural compounds with less pronounced side effects seems relevant.

Natural compounds of marine origin including triterpene glycosides from holothurians attract attention as substances possessing various types of biological activity. It was previously established that a number of triterpene glycosides from sea cucumbers of the genus *Cucumaria*, cucumarioside A_2_-2 and frondoside A in particular exhibit a pronounced antitumor effect in vitro and in vivo; block cell proliferation, DNA biosynthesis and the cell cycle, induce apoptosis and inhibit colony formation of tumor cells [3,4,5,6]. Using the standard Calcein-AM efflux assay only, it has recently been demonstrated that the same triterpene glycosides are able to increase the accumulation of fluorescent probe in tumor cells probably due to MDR inhibition [7,8]. However, the effect of glycosides on magnification of sensitivity of cancer cells to antitumor compounds by MDR blocking has not been shown. In this regard, the aim of our work was to study the effect of triterpene glycoside, cucumarioside A_2_-2, on the blocking of multidrug resistance of mouse Ehrlich ascites carcinoma cells in more detail, including glycoside effects upon cytostatic drug, doxorubicin, accumulation, efflux and cytotoxicity against cancer cells in vitro, as well as the combined antitumor effect of glycoside and doxorubicin using mice bearing Ehrlich carcinoma in vivo.

## 2. Results

### 2.1. Сucumarioside A_2_-2 Increases the Amount of Calcein AM and Doxorubicin in Tumor EAC Cells

**Figure 1:**
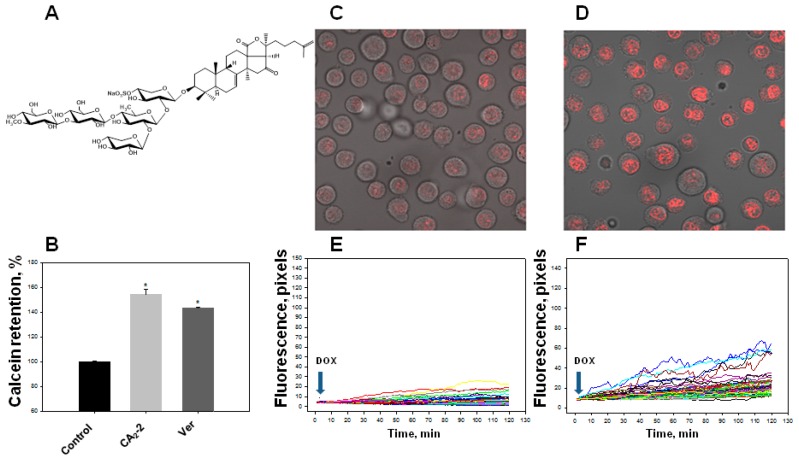
Effect of cucumarioside A_2_-2 (CA_2_-2) on the accumulation of a Calcein-AM and doxorubicin (DOX) in Ehrlich ascites carcinoma (EAC) cells. Chemical structure of CA_2_-2 (**A**). Effect of CA_2_-2 (0.1 μM) and verapamil (Ver, 0.001 μg/mL) on the accumulation of a Calcein-AM (**B**). Accumulation of DOX in the cell nuclei. Images obtained 2 h after incubation with DOX: Control cells (**C**) and cells pre-exposed with CA_2_-2 (**D**). The effect of CA_2_-2 (0.01 μM) on the dynamics of DOX accumulation in the nuclei of EAC cells: Control cells (**E**) and cells pre-exposed with CA_2_-2 (**F**). Records of changes in the fluorescence of the nuclei of individual cells in a monolayer are shown. Arrows indicate the time of DOX administration. * *p* < 0.05.

We found that the studied triterpene glycoside CA_2_-2 effectively blocks the activity of P-gp at a concentration of 0.1 μM resulting in an increase in the quantity of Calcein probe in the cytoplasm. Blocking MDR in EAC cells and an increase in the fluorescence intensity of the Calcein probe accumulated in the cytoplasm amounted to about 50% as compared to control level similarly to standard MDR inhibitor, verapamil (Figure 1B).

**Figure 2:**
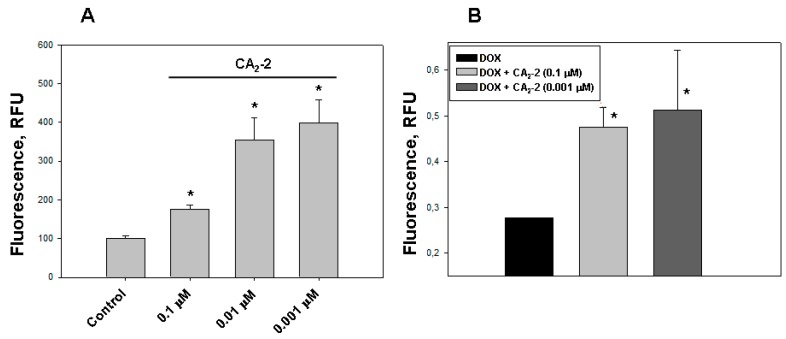
The effect of CA_2_-2 on the accumulation and efflux of DOX in EAC cells. The content of DOX in the cell nuclei after its accumulation within 2 h (**A**) and its residual amount in the nuclei 1 hour after washing the cells (**B**) are shown. * *p* < 0.05.

Using confocal microscopy, we found that the incubation of EAC cells with CA_2_-2 in the concentration range of 0.001–0.1 μM for 2 h leads to a noticeable increase in the dynamics of doxorubicin uptake and its accumulation in the nuclei of tumor cells. Figure 1C–F clearly shows that after 2 h from the beginning of the experiment, the final concentration of doxorubicin accumulated in the nuclei is significantly higher in the cells treated with CA_2_-2 compared with the control untreated cells. An inversely proportional dependence of the MDR-inhibiting effect on glycoside concentration was noted. It was shown that preliminary incubation of the cells with CA_2_-2 at a concentration of 0.001 μM led to maximum accumulation of doxorubicin in the nuclei of EAC cells (Figure 2A).

The study of DOX release from tumor cells was performed using spectrofluorimetry. We found that the nanomolar concentrations of CA_2_-2 are able to delay the efflux of DOX from the cytoplasm of tumor cells after its initial accumulation in the nuclei. A noticeable effect of inhibition was observed at a concentration of glycoside of 0.001 and 0.1 μM after preliminary incubation of cancer cells with glycoside and subsequent accumulation of DOX in EAC cells for 2 h. The greatest effect of delaying the release of fluorescent antitumor antibiotic was observed 1 h after washing the cells at a concentration of glycoside of 0.001 μM (Figure 2B).

### 2.2. Cucumarioside A_2_-2 Enhances the Cytotoxic Activity of Doxorubicin in Vitro

**Figure 3:**
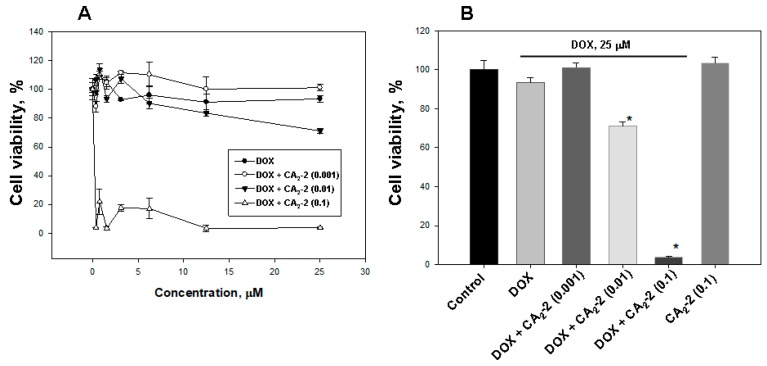
The effect of CA_2_-2 on the cytotoxic activity of DOX (**A**) against EAC cells, evaluated by MTT. Cytotoxic activity of DOX (25 μM) and various concentrations of CA_2_-2 (**B**). Cells were pre-exposed with CA_2_-2 for 2 h and than incubated with DOX for the next 48 h. * *p* < 0.05.

The studying of the influence of CA_2_-2 on DOX cytotoxic activity showed that glycoside at a concentration of 0.1 and 0.01 μM significantly increases DOX cytoxicity (Figure 3A,B). The greatest effect was found at a concentration of 0.1 μM, which decreases cell viability to more than 80%, compared with DOX alone. The glycoside concentration of 0.01 μM was shown to inhibit cell viability up to around 25%, and the minimal test concentration of 0.001 μM did not affect the cytotoxic activity of DOX. At the same time, CA_2_-2 at a concentration of 0.1 μM, as well as doxorubicin at a concentration of 25 μM did not exhibit cytotoxic activity by itself (Figure 3B).

### 2.3. Сucumarioside A_2_-2 Increases the Survival of Mice with Ehrlich Ascites Carcinoma Treated with Doxorubicin

**Figure 4:**
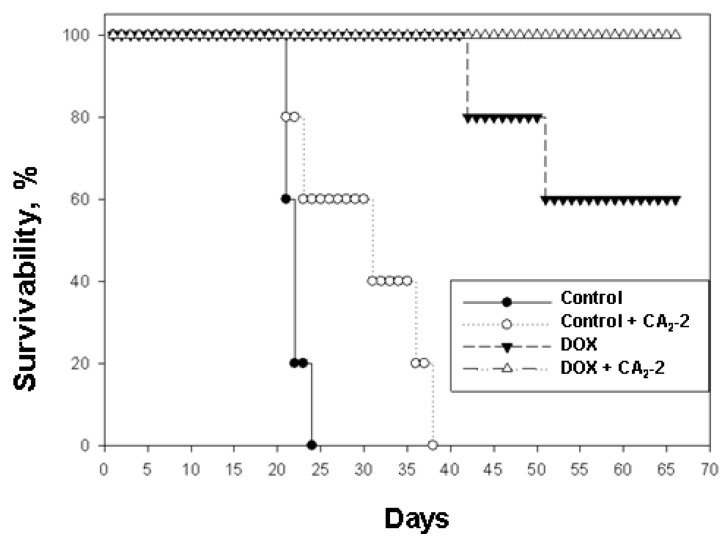
The effect of cucumarioside A_2_-2 on the antitumor activity of doxorubicin in the in vivo model of mouse Ehrlich ascites carcinoma.

It was found that the survival time of control animals implanted with untreated cancer cells was less than that of animals inoculated with the cells obtained from CA_2_-2 treated animals. In the first control group, group 1, an average life span (ALS) of 21.5 days was observed. In the second group of control animals subjected to in vivo exposure to CA_2_-2, which then received only physiological saline, an increase in the ALS was indicated. Thus, in group 2 the last animal died on the 36th day while in the control group 1, on the 23rd day of the experiment. So, ALS of mice in group 2 was 29.8 days (Table 1, Figure 4).

When using DOX at a dose of 2 mg/kg, a significant increase in life expectancy was observed (group 3). The first mouse in this group died only on the 42nd day after tumor inoculation, the second mouse fell on the 52nd day, and the other animals in the group remained alive until the 70th day of observation. ALS in this group was 60.8 days, and animal survivability by day 70 was 60%. In group 4 the animals received tumor cells from mice previously exposed to CA_2_-2 in vivo and then subsequently were treated with DOX, the survival rate was 100% for the entire observation period of 70 days (Table 1, Figure 4).

## 3. Discussion

Doxorubicin is a well-known medicinal drug used for therapy of various types of tumors. It is an antitumor antibiotic of the anthracycline series. It has an antimitotic and antiproliferative effect. The mechanism of the antitumor effect of DOX is the interaction with DNA, the formation of free radicals and the suppression of nucleic acid synthesis. One of the noted drawbacks of using doxorubicin is the emergence in tumor cells resistant to this cytostatic as a result of activation of the multidrug resistance mechanism. At the same time, it was shown that a number of compounds (for example, calcium channel blockers verapamil and diltiazem) can enhance the antitumor effect of doxorubicin by suppressing MDR of tumor cells [9].

It is known that Ehrlich ascites carcinoma cells exhibit multidrug resistance phenomenon due to overexpression of P-glycoprotein in response to the action of a number of antitumor compounds [10,11]. We have found that incubation of Ehrlich ascites carcinoma cells with the holothurian triterpene glycoside, cucumarioside A_2_-2, leads to increasing doxorubicin accumulation, blocking the release of doxorubicin and increase in its retention in mouse tumor cells. Despite the fact that the rate of DOX uptake in tumor cells pre-exposed with different concentrations of CA_2_-2 was different, the final amount of doxorubicin remaining in the cells was approximately the same. This leads to a significant increase in the cytotoxic activity of doxorubicin against tumor cells in vitro. This effect can be directly related to the ability of CA_2_-2 to block MDR, since CA_2_-2 alone did not exhibit a cytotoxic effect. This fact can be confirmed by the results of the assessment of blocking of Calcein-AM release from tumor cells, which are the standard experimental approach to search for MDR blockers [12]. 

In addition, we have shown that in vivo pre-treatment of mice with Ehrlich ascites carcinoma by CA_2_-2 followed by transplantation of such treated tumor cells into intact animals and subsequent treatment of these mice with doxorubicin significantly increases the antitumor activity of doxorubicin in vivo. This effect can be also attributed to the blocking of MDR in EAC cells, which results in the increasing of tumor cell sensitivity to cytostatics owing to their increased accumulation in cells.

Among triterpenoids of marine origin, substances were found that possess various types of biological activity, including the selective cytotoxic activity against MDR cells, as well as the blocking of P-gp. Thus, sipholenol A isolated from the Red Sea sponge *Callyspongia (Siphonochalina) siphonella* significantly eliminated the resistance of P-gp overexpressing KB-C2 cells to colchicine. Sipholenol A did not act on KB-3-1 epidermoid drug-sensitive carcinoma cells (which do not express P-gp) and on MRP1-expressing KB-CV60 cells, so the researchers suggested that this triterpenoid is selective for P-gp and consider the sipholenol A as a potential P-pg modulator [13]. 

Later, three new sipholan triterpenoids were isolated from the same sponge, sipholenone E, sipholenol L and siphonellinol D, and studied as potential inhibitors of P-glycoprotein. These compounds enhanced the cytotoxic activity of several well-known anticancer drugs including colchicine, vinblastine and paclitaxel, which are substrates for P-gp, significantly increasing the sensitivity of cancer cells with a P-gp overexpressed phenotype (KB-C2) in a dose-dependent manner. At the same time, these sipholan triterpenoids did not affect the sensitivity of KB-3-1 and KB-C2 cells to cisplatin, a chemotherapeutic drug that is not a substrate for P-gp [14].

Recently, during the screening assay it was found that the holothurian triterpene glycosides, cucumarioside A_2_-2 from *Cucumaria japonica* and frondoside A from *C. frondosa*, as well as their complexes with cholesterol, are able to effectively block MDR and prevent the release of low molecular weight fluorescent agent Calcein-AM from tumor cells [7,8]. Besides, the new medicinal preparation, Cumaside created on the basis of the triterpene glycoside, cucumarioside A_2_-2, led to a synergistic effect and enhanced the antitumor effect of the cytostatic drug 5-fluorouracil in combined antitumor therapy, which can also be a manifestation of MDR blocking [15].

It is well known that the membranotropic properties of holothurians triterpene glycosides such as hemolytic, cytotoxic or immunostimulatory activity largely depends on the presence of carbohydrate chain in the molecule and especially the presence of a number of sulphate groups in carbohydrate moieties. Usually, aglycone alone is completely inactive [16,17]. In the very near future, we plan to investigate the ability of triterpene glycosides with different carbohydrate chains and aglycones to block MDR in various types of tumor cells.

Thus, it was shown that a number of triterpene compounds isolated from marine organisms are able to block MDR in tumor cells and inhibit the growth of tumor cells that are resistant to antitumor agents. Continuation of research in this direction may lead to the creation of a new natural remedy based on holothurian triterpene glycosides that suppresses the activity of P-glycoprotein in tumor cells, and its further use as a medicinal drug for combined antitumor therapy together with known cytostatics.

## 4. Materials and Methods 

### 4.1. Drugs 

Monosulfated triterpene glycoside cucumarioside А_2_-2 (CA_2_-2) was isolated from the sea cucumber *C. japonica* using standard procedures [18]. The purity of glycoside was checked by the ^13^С NMR. The chemical formula of CA_2_-2 is presented in Figure 1A. Water solution of CA_2_-2 was used in all experiments. Commercially available doxorubicin (DOX) was purchased from Pharmachemi BV (Netherlands) and dissolved in phosphate buffer saline (PBS).

### 4.2. Animals and Cells 

CD-1 mice weighing 18–20 g were purchased from RAMS ‘Stolbovaya’ nursery (Russia) and kept at the animal facility in standard conditions. All experiments were conducted in compliance with all of the rules and international recommendations of the European Convention for the Protection of Vertebrate Animals Used for Experimental Studies. 

The museum tetraploid strain of mouse Ehrlich ascites carcinoma (EAC) was provided by the N.N. Blokhin Russian Oncology Center (Moscow, Russia). EAC cells were injected into the peritoneal cavity of CD-1 mice. Cells for experiments were collected 7 days after inoculation. For this purpose mice were killed by cervical dislocation, and the ascitic fluid containing tumor cells was collected with a syringe. The cells were washed two times by centrifugation at 2000 rpm (450× *g*) for 10 min in PBS (pH 7.4) followed by resuspension in RPMI-1640 culture medium without serum. The cell number and viability were determined with a hemocytometer and the trypan blue staining procedure. The final cell concentration in the media was usually 2–5 × 10^6^ cells/mL.

### 4.3. Calcein-AM Efflux Assay 

The experiments were initiated by washing the cells with PBS. Cells were treated with different concentration of CA_2_-2 at 37 °C for 30 min. Then 0.25 μM of calcein-AM (Molecular Probes, USA) was added to each well. After 15 min incubation at 37 °C, cells were washed twice with PBS and splitted to quantify P-gp activity by fluorescence in a microplate reader (Fluoroscan Accent, Finland) at λex = 494 nm and λem = 517 nm [12]. Verapamil (Sigma, USA) was used as a positive control.

### 4.4. MTT Viability Assay 

The cytotoxic activity of doxorubicin and the combined effect of doxorubicin and cucumarioside A_2_-2 were evaluated using the MTT method. For this purpose, 5 × 10^4^ cells/well were seeded in 96-well microplates, then 20 μL of DOX solution at different concentrations was added and microplates were incubated for 48 h in a CO_2_-incubator at 37 °C and 5% of CO_2._ To study the combined effect, 20 μL of cucumarioside A_2_-2 solution at different concentrations was added to wells first, and EAC cells were incubated during 2 h for blocking of MDR. Then 20 μL of DOX (25 μM final concentration) was added, and cells were incubated for 48 h at 37 °C and 5% of CO_2_. Then the medium was replaced with 100 μL of fresh medium containing 10 μL of MTT solution (Sigma, USA) and microplates were incubated for another 4 h. After that 100 μL SDS-HCl solution was added and microplates were incubated again at 37 °C for 4–18 h. The optical density was measured with a microplate spectrophotometer Multiskan FC (Thermo Scientific, Canada) at 570 nm. The cytotoxic activity of the substances was expressed as percent of cell viability in comparison with the control.

### 4.5. Measurement of Doxorubicin Accumulation and Efflux 

The measurement of DOX accumulation in cells was performed according to the method in [19]. Cells were seeded on microscope imaging chambers at a concentration of 2 × 10^6^ cells/mL. Then the chambers were placed on the microscope stage. To study the effect of glycoside on the time course of DOX accumulation, EAC cells were pre-exposed to different concentrations of CA_2_-2 for 30 min before being mounted in the microscope stage. DOX at a final concentration of 5 μM were added to the cells and serial images at 1-min intervals were collected for 2 h and analyzed using a confocal microscope Axiovert 200M LSM510 META (Carl Zeiss, Germany). Doxorubicin fluorescence was excited with an argon laser at 488 nm, and the emission was collected through a 505-nm long-pass filter. Post-data acquisition image analysis was performed using LSM 510 software release version 4.2 (Carl Zeiss, Germany). Cell images were analyzed as mean DOX fluorescent intensity per pixel in a region of interest (nuclei). Results were obtained from analyzing images from three to five experiments. 

To study of doxorubicin efflux (retention), EAC cells were seeded to a 24-well plate. Different concentrations of CA_2_-2 were added to each well and plates were incubated at 37 °C for 30 min to block MDR. Then, DOX (5 μM, final concentration) was added and plates were incubated for 2 h at 37 °C to accumulate doxorubicin in the cells. After a certain time, aliquots of 100 μL of the cell suspension were transferred into a 1.5 mL Eppendorf tube, pelleted by centrifugation, and washed twice with cold PBS. Then 100 μL of cell suspension was replaced with a black 96-well microplate and the fluorescence of doxorubicin was measured using a Fluoroscan Accent (Finland) plate reader at λex = 485 nm and λem = 620 nm.

### 4.6. Study of Antitumor Activity of Doxorubicin in Vivo 

The influence of CA_2_-2 on the antitumor activity of DOX was assessed using the method in [20] with minor modifications. The tumors were maintained by intraperitoneal transplantation of 2 × 10^6^ cells/mouse. Mice were treated with CA_2_-2 in a single dose of 10 μg/kg (intraperitoneal injection, *i.p.*) in a volume of 0.5 mL once a day for 5 days. The control group of mice received the same volume of saline. On the 7th day an equal amount of cancer cells from either CA_2_-2 pre-treated mice or from animals without pre-treatment were isolated and injected *i.p.* into fresh mice. Doxorubicin treatment (2 mg/kg *i.p.*) was started 24 h after tumor transplantation every other day only three times. Four groups of animals were formed with 5 mice in the group:

Group 1—control. The intact tumor cells were transplanted to animals. Then mice were treated with saline;

Group 2—control. The CA_2_-2 treated tumor cells were transplanted to animals. Then mice were treated with saline;

Group 3—experimental. The intact tumor cells were transplanted to animals. Then mice were treated with DOX;

Group 4—experimental. The CA_2_-2 treated tumor cells were transplanted to animals. Then mice were treated with DOX.

Observation of the animals was continued for 70 days. The average life span (ALS) for mice bearing tumors was determined for all groups of animals. 

### 4.7. Statistics 

All data are expressed as mean ± S.E. from three or more experiments, and they were statistically evaluated by Student’s *t*-test. Differences were considered significant when *p* < 0.05.

## Figures and Tables

**Table 1:** The effect of cucumarioside A_2_-2 and doxorubicin on the survivability of mice with Ehrlich ascites carcinoma (inoculation of 2.0 × 10^6^ cells per animal).

Group of Animals	CA_2_-2, dose	DOX, dose	ALS, days
1, Control	-	-	21.5 ± 0.4
2, Control + CA_2_-2	10 μg/kg/5 days *i.p.*	-	29.8 ± 3.2
3, DOX	-	2 mg/kg/3 days *i.p.*	60.8 ± 5.9
4, DOX + CA_2_-2	10 μg/kg/5 days *i.p.*	2 mg/kg/3 days *i.p.*	70.0 ± 0.0
